# Post-Treatment Surveillance for Lymphatic Filariasis in Plateau and Nasarawa States, Nigeria: Results of Transmission Assessment Surveys

**DOI:** 10.4269/ajtmh.20-0020

**Published:** 2020-03-30

**Authors:** Abel Eigege, Gregory S. Noland, Solomon E. Adelamo, Kenrick Nwodu, Adamu Sallau, John Umaru, Bulus S. Mancha, Emmanuel Davies, Jacob Danboyi, Jonathan A. Kadimbo, Yisa A. Saka, Ifeoma Anagbogu, Emmanuel S. Miri, Frank O. Richards

**Affiliations:** 1 The Carter Center, Jos, Nigeria;; 2 The Carter Center, Atlanta, Georgia;; 3 Federal Ministry of Health, Abuja, Nigeria;; 4 State Ministry of Health, Lafia, Nasarawa;; 5 State Ministry of Health, Jos, Plateau

## Abstract

Following the halt of mass drug administration (MDA) for lymphatic filariasis (LF), the WHO recommends at least 4 years of post-treatment surveillance (PTS) to confirm that transmission recrudescence or importation does not occur. The primary means of evaluation during PTS is repeated transmission assessment surveys (TASs) conducted at 2- to 3-year intervals after TAS-1 stop-MDA surveys. This study reports the results of TAS-2 and TAS-3 surveys in Plateau and Nasarawa states (pop. 6.9 million) of Nigeria divided into a minimum of seven evaluation units (EUs) per TAS. A total of 26,536 first- and second-year primary school children (approximately 6–7 years old) were tested for circulating filarial antigen (CFA) between 2014 and 2017. Of 12,313 children tested in TAS-2 surveys, only five (0.04%) were CFA positive, with no more than two positive samples from any one EU, which was below the critical value of 20 per EU. Of 14,240 children tested in TAS-3 surveys, none (0%) were CFA positive. These results indicate that LF transmission remains below sustainable transmission levels and suggest that elimination of transmission has been achieved in Plateau and Nasarawa, Nigeria.

## INTRODUCTION

Lymphatic filariasis (LF) is a mosquito-transmitted parasitic disease caused by infection with *Wuchereria bancrofti, Brugia malayi*, or *Brugia timori*. It is a leading cause of permanent and long-term disability due to its disfiguring and debilitating manifestations that include lymphedema, elephantiasis, male scrotal swelling (hydrocele), and acute febrile episodes of adenolymphangitis.^1^ These conditions result from chronic dysfunction caused by adult worms inhabiting the lymphatic vessels. Gravid females produce millions of infective microfilariae (mf) that circulate in the lymphatic and blood systems. Treatment with albendazole (donated by GlaxoSmithKline, Brentford, United Kingdom) plus either ivermectin (Mectizan^®^, donated by Merck, Kenilworth, NJ) or diethylcarbamazine (DEC, donated by Eisai, Tokyo, Japan) reduces the number of mf in circulation, thereby preventing transmission to mosquitoes.^2^ Annual mass drug administration (MDA) at sufficient population coverage (≥ 65%) is predicted to interrupt LF transmission in a population in 4–6 years—the estimated lifespan of adult worms.^3^

The WHO targets the elimination of LF as a public health problem by 2020 through a dual strategy of MDA to interrupt transmission and morbidity management and disability prevention (MMDP) to care for those already suffering from LF.^4^ When WHO launched the Global Program to Eliminate LF (GPELF) in 2000, nearly 1.1 billion people in 80 countries were at risk of infection.^1^ Nigeria bears the largest LF burden in Africa and second largest burden globally after India with 120 million at risk and approximately 71% of districts, called local government areas (LGAs), considered endemic.^4^ To demonstrate the feasibility of interrupting LF transmission in high-burden areas, the Nigerian Federal Ministry of Health (FMOH), with assistance from The Carter Center, established an LF elimination program in 1997 in Plateau and Nasarawa states of north-central Nigeria. The program capitalized on the onchocerciasis (river blindness) community-directed treatment with ivermectin program that operated in 12 hyper-/meso-endemic LGAs of the two-state area since 1992.^5^

Baseline LF mapping conducted in 1998–2000 revealed all 30 LGAs of Plateau and Nasarawa were endemic with mean LF antigen prevalence of 23% (range 4–62%) in adults.^6^ Annual albendazole–ivermectin MDA started in 2000 in two LGAs; full geographic coverage of all 30 LGAs (each LGA considered an implementation unit [IU]) was achieved in 2003 (Table 1). In 2007–2008, after a minimum of 5 years of MDA for LF, stop-MDA surveys were conducted using the WHO Pacific regional program to eliminate LF (PacELF) “C-survey” methodology as an alternative to the complex multi-survey strategy recommended by WHO at that time.^7,8^ Ten of the 30 LGAs met the “C-survey” stop-MDA criterion of antigen prevalence less than 2% at the 95% confidence limit among individuals older than 2 years.^9^ One LGA that qualified to stop MDA, Jos South, opted to continue annual treatments because of high rates of focal infections in a pre-survey spot-check site. Of the remaining nine qualifying LGAs, four halted albendazole–ivermectin MDA in 2010, whereas five halted albendazole distribution but continued ivermectin MDA for onchocerciasis elimination. In addition, one LGA with a borderline failure result by PacELF criteria, Barkin Ladi, was permitted to stop LF MDA because antigen prevalence among children aged 3–9 years was significantly less than 2% (point estimate = 0%; 95% upper confidence limit = 1.2%). In total, 20 LGAs continued annual LF MDA through 2012, with average reported annual eligible population treatment coverage of 93% over a maximum of 13 rounds of annual MDA (range 83%–103%). In 2012, stop-MDA transmission assessment surveys (TAS-1) determined that antigen prevalence among children aged 6–7 years in the 20 LGAs, configured into four evaluation units (EUs) was significantly below 2%.^10^ This result, combined with failure to detect any infected mosquitoes from sentinel village xenomonitoring throughout the two-state area in 2011–2012,^11^ led to the halt of MDA for LF across all of Plateau and Nasarawa after 2012. Annual ivermectin treatments continued, however, in the 12 onchocerciasis hyper-/meso-endemic LGAs until 2017.^12^

**Table 1:** MDA treatment summary for LF and RB, and baseline LF CFA prevalence for the 30 LGAs of Plateau and Nasarawa states, Nigeria, by transmission assessment survey EU

EU	State	LGAs	Population (2015 est).	Baseline LF CFA prevalence in adults, 1998–2000 (%)	Years of LF MDA	Number of LF MDA rounds	Years of RB MDA
1a	Plateau	Jos North	570,004	4	2003–2009	7	
		Langtang South	141,147	20	2003–2009	7	
			**711,151**	**12.0**			
1b	Nasarawa	Keffi	106,977	11	2003–2009	7	
		Keana	91,606	14	2003–2009	7	
			**198,583**	**12.5**			
2	Plateau	Barkin Ladi	232,711	18	2003–2009	7	
		Jos South	407,243	11	2002–2012	11	
		Langtang North	186,739	19	2002–2012	11	
		Mangu	391,596	27	2002–2012	11	
		Pankshin*	254,522	41	2000–2012	13	1993–2017
		Quan-Paan	261,473	32	2002–2012	11	
		Riyom	174,675	19	2002–2012	11	
			**1,908,960**	**23.9**			
3	Plateau	Kanam	220,272	44	2002–2012	11	
		Kanke*	161,221	58	2001–2012	12	1993–2017
		Mikang	129,338	25	2003–2012	10	
		Shendam	276,195	22	2003–2012	10	
		Wase	214,716	24	2002–2012	11	
			**1,001,742**	**34.6**			
4	Plateau	Bassa*	248,103	15	2001–2009	9	1993–2017
		Bokkos*	236,943	11	2001–2009	9	1993–2017
		Jos East*	113,658	16	2001–2009	9	1993–2017
			**598,704**	**14.0**			
5	Nasarawa	Awe	136,015	8	2003–2012	10	
		Lafia*	626,321	34	2002–2012	11	1993–2017
		Nasarawa Eggon*	222,967	25	2001–2012	12	1993–2017
		Toto*	183,396	13	2001–2012	12	1993–2017
		Wamba*	120,660	34	2001–2012	12	1993–2017
			**1,289,358**	**22.8**			
6	Nasarawa	Akwanga*	250,281	29	2000–2012	13	1993–2017
		Doma	124,959	34	2002–2012	11	
		Nasarawa	256,852	10	2002–2012	11	
		Obi	147,407	38	2002–2012	11	
			**779,500**	**27.8**			
7	Nasarawa	Karu*	305,338	21	2001–2009	9	1993–2017
		Kokona*	109,645	9	2001–2009	9	1993–2017
			**414,983**	**15.0**			

CFA = circulating filarial antigen; EU = evaluation unit; LF = lymphatic filariasis; LGA = local government area; MDA = mass drug administration; RB = river blindness.

* Onchocerciasis hyper-/meso-endemic.

WHO recommends at least 4 years of post-treatment surveillance (PTS) following the halt of MDA to confirm that LF transmission recrudescence or importation does not occur.^3^ A primary strategy for PTS includes repeated cross-sectional TASs 2 years (TAS-2) and 4 years (TAS-3) after TAS-1 or an equivalent stop-MDA survey.^3^ This report describes results from TAS-2 and TAS-3 PTS surveys across Plateau and Nasarawa states.

## METHODS

### Study area and survey design.

**Figure 1.**
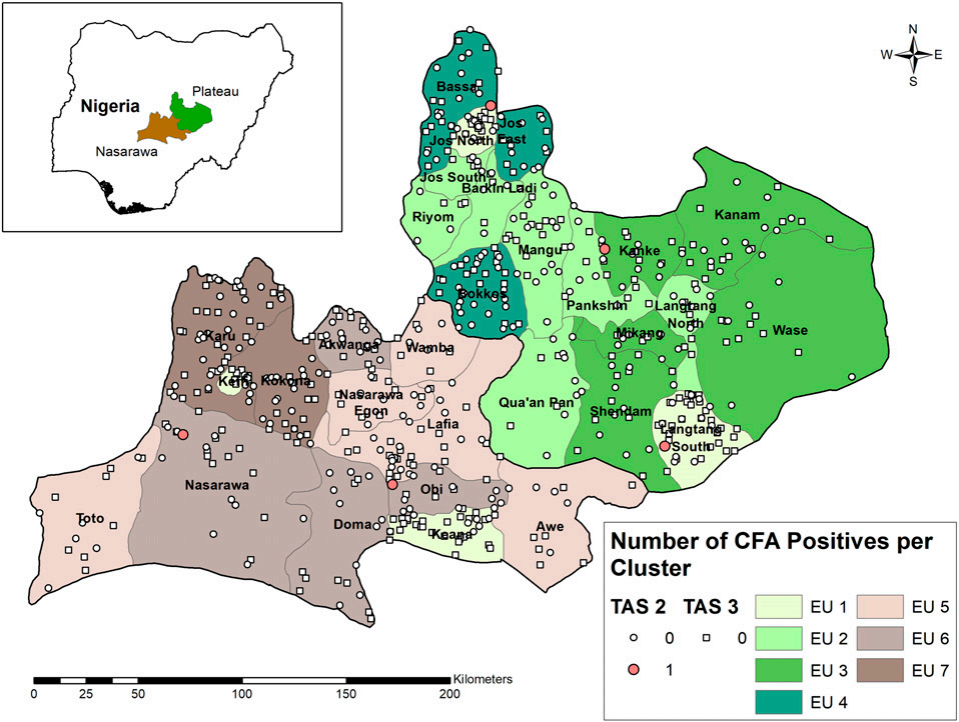
Lymphatic filariasis circulating filarial antigen prevalence, by cluster, from school-based transmission assessment surveys (TAS-2, circle; TAS-3, square) in Plateau and Nasarawa states, Nigeria, 2014–2017. This figure appears in color at www.ajtmh.org.

Cross-sectional school-based cluster surveys were conducted across all 30 LGAs of Plateau (2015 population est. 4.2 million) and Nasarawa (2.7 million) states in 2014–2015 (TAS-2) and 2016–2017 (TAS-3). Evaluation units were formed based on chronology of TAS eligibility, geography, and epidemiological similarity. Transmission assessment survey guidelines permit the aggregation of multiple, noncontiguous IUs into an EU if the IUs share similar epidemiologic features and have completed at least five effective rounds of MDA.^3^ Three general groups of EUs were formed (Figure 1). The first group included the four LGAs that stopped LF MDA in 2010 (Jos North and Langtang South in Plateau, and Keffi and Keana in Nasarawa). These four were combined as one EU (EU1) for TAS-2 in 2014 but split into two EUs (EU1a and 1b; EU1 per state) for TAS-3 in 2016. The second group included the 21 LGAs assessed by TAS-1 in 2012.^10^ The same four-EU configuration of TAS-1 was retained for TAS-2 in 2015 and TAS-3 in 2017: Barkin Ladi, Jos South, Langtang North, Mangu, Pankshin, Quan-Paan, and Riyom (EU2), and Kanam, Kanke, Mikang, Shendam, and Wase (EU3) in Plateau; Awe, Lafia, Nasarawa Eggon, Toto, and Wamba (EU5), and Akwanga, Doma, Nasarawa, and Obi (EU6) in Nasarawa. The third group comprised the onchocerciasis co-endemic LGAs that halted albendazole treatment in 2010 but continued ivermectin treatment until 2017: Bassa, Bokkos, and Jos East in Plateau (EU4) and Karu and Kokona in Nasarawa state (EU7). In accordance with TAS guidelines, the population size in each EU was less than two million and the estimated target population (6–7-year-old children) was greater than 50,000.

### Sample sizes.

The target sample size per EUs ranged from 1,548 to 1,692 with critical cutoff values of 18–20 antigen-positive children (Tables 2 and 3). Transmission assessment survey sample sizes and critical cutoff values are powered so that an EU has at least a 75% chance of passing if the true antigen prevalence is 1.0% and no more than about a 5% chance of passing (incorrectly) if the true antigen prevalence is ≥ 2.0%—the level below which *Anopheles-*transmitted *W. bancrofti* is believed to be unsustainable.^3^

**Table 2:** Summary of TAS-2 results by EU in 30 LGAs of Plateau and Nasarawa states, Nigeria

Year	EU	State	LGAs	Number of schools sampled	Target sample size	Number of children tested	Test	Number of positive (%)	TAS critical cutoff	TAS result (pass/fail)
2014	1	Plateau and Nasarawa	Jos North, Langtang South, Keffi, and Keana	37	1,692	1,759	ICT	2 (0.11)	20	Pass
2015	2	Plateau	Barkin Ladi, Jos South, Langtang North, Mangu, Pankshin*, Quan-Paan, and Riyom	43	1,692	1,731	ICT	1 (0.06)	20	Pass
2015	3	Plateau	Kanam, Kanke*, Mikang, Shendam, and Wase	40	1,692	1,744	ICT	0 (0.0)	20	Pass
2015	4	Plateau	Bassa*, Bokkos*, and Jos East*	46	1,684	1,737	ICT	0 (0.0)	20	Pass
2015	5	Nasarawa	Awe, Lafia*, Nasarawa Eggon*, Toto*, and Wamba*	40	1,692	1,721	ICT	0 (0.0)	20	Pass
2015	6	Nasarawa	Akwanga*, Doma, Nasarawa, and Obi	43	1,692	1,714	ICT	2 (0.12)	20	Pass
2015	7	Nasarawa	Karu* and Kokona*	42	1,692	1,900	ICT	0 (0.0)	20	Pass
Totals				291	11,836	12,306	–	5 (0.04)	–	–

EU = evaluation unit; ICT = immunochromatographic test; LGA = local government area; TAS = transmission assessment survey.

* Onchocerciasis hyper-/meso-endemic.

**Table 3:** Summary of TAS-3 results by EU in 30 LGAs of Plateau and Nasarawa states, Nigeria

Year	EU	State	LGAs	Number of schools sampled	Target sample size	Number of children tested	Test	Number of positive results (%)	TAS critical cutoff	TAS result (pass/fail)
2016	1a	Plateau	Jos North and Langtang South	44	1,692	1,796	ICT	0	20	Pass
2016	1b	Nasarawa	Keffi and Keana	40	1,548	1,545	FTS	0	18	Pass
2017	2	Plateau	Barkin Ladi, Jos South, Langtang North, Mangu, Pankshin*, Quan-Paan, and Riyom	41	1,692	1,965	FTS	0	20	Pass
2017	3	Plateau	Kanam, Kanke*, Mikang, Shendam, and Wase	44	1,692	2,034	FTS	0	20	Pass
2017	4	Plateau	Bassa*, Bokkos*, and Jos East*	40	1,684	1,726	FTS	0	20	Pass
2017	5	Nasarawa	Awe, Lafia*, Nasarawa Eggon*, Toto*, and Wamba*	45	1,692	1,825	FTS	0	20	Pass
2017	6	Nasarawa	Akwanga*, Doma, Nasarawa, and Obi	45	1,692	1,734	FTS	0	20	Pass
2017	7	Nasarawa	Karu* and Kokona*	42	1,556	1,605	FTS	0	18	Pass
Totals				341	13,248	14,230		0		

EU = evaluation unit; FTS = filariasis test strip; ICT = immunochromatographic test; LGA = local government area; TAS = transmission assessment survey.

* Onchocerciasis hyper-/meso-endemic.

School-based TASs were implemented because of high enrollment rates (> 75%) in the survey area. Within each EU, 45 schools (slightly fewer in smaller EUs) were selected by interval (systematic) selection following a random start from an ordered list of Ministry of Education–registered schools. This process was repeated for each TAS. For surveys conducted from 2014 to 2016, approximately 45 first- and second-year primary school children (around 6–7 years old) were randomly selected at each school with a maximum of 55 children from any one school. In 2017, a fixed proportion of children at each school (80%) were selected for enrollment to enable equal probabilities of selection across the EU.

### Blood testing.

Finger-prick blood samples were collected by certified laboratory scientists from all assenting children and tested on-site for the presence of circulating filarial antigen (CFA) by BinaxNOW Filariasis immunochromatographic test (Alere Inc., Scarborough, ME) or Filariasis Test Strip (Alere, Inc.) according to the manufacturer’s instructions. Results were read at 10 minutes, recorded on paper forms, and communicated confidentially to each child as well as to the school headmaster. Geocoordinates were measured by Samsung Galaxy Tab tablets. All antigen-positive children were followed-up for night blood smear testing and then offered albendazole–ivermectin according to FMOH guidelines. Performance of rapid tests was validated before and after the field work with positive controls (provided by NIH/NIAID Filariasis Research Reagent Resource Center).

### Ethical approval and consent procedures.

The surveys were approved by the Nigerian National Health Research Ethics Committee (approval numbers NHREC/01/01/2007-17/04/2014, NHREC/01/01/2007-20/04/2015, NHREC/01/01/2007-10/02/2016, and NHREC/01/01/2007-18/04/2017) and also considered as public health nonhuman subjects research activities by the Emory University Institutional Review Board. Participation in the surveys was voluntary. Individual oral assent was obtained from selected students and written informed consent obtained from a parent or guardian.

## RESULTS

For TAS-2 surveys, a total of 12,352 children were selected for enrollment in 291 schools across seven EUs. A total of 12,306 children were tested (0.4% refusal rate), with the target sample size exceeded in each EU (range 1,714–1,907) (Table 2). Five children (0.04%) were CFA positive. The number of CFA-positive individuals in each EU (range 0–2) was less than the cutoff of 20, meaning that each EU “passed” TAS-2. Geographically, the five positive samples were widely separated (Figure 1), with no single school or LGA having more than one positive individual. None of the five individuals were mf positive in follow-up night blood smear examination.

A total of 14,240 children were tested in 341 schools across eight EUs in TAS-3 surveys (0.5% refusal rate). No CFA-positive samples were identified, meaning that each EU “passed” TAS-3.

## DISCUSSION

Since the launch of the GPELF in 2000, approximately 7.1 billion treatments have been distributed resulting in the elimination of LF as a public health problem in 11 countries through 2017.^13^ However, progress has been slowest in Africa, where only two African countries to date—Togo and Egypt—have met this milestone.^13^ Validation by WHO requires that a country should meet a combination of an epidemiological criterion (infection prevalence significantly lower than putative sustainable transmission levels for at least 4 years of PTS) and demonstration of the availability of care for LF patients.^14^ Results from this study indicate that two formerly endemic Nigerian states, Plateau and Nasarawa, have met the WHO epidemiological requirements for PTS—that is, < 2% antigen prevalence in children in areas where *W. bancrofti* is transmitted by *Anopheles* or *Culex* mosquitoes—and furthermore suggest that LF transmission has been eliminated in the area.

The reduction in antigen prevalence in these areas from an average of 23% among adults at baseline to zero CFA-positive children in TAS-3 demonstrates the efficacy of high-coverage MDA (93% average reported coverage for all MDA rounds in the area) complimented by the protective benefits of bed nets.^15,16^ Approximately, eight million insecticide-treated nets (ITNs) or long-lasting insecticidal nets (LLINs) have been distributed to date in Plateau and Nasarawa through mass distributions and other channels supported by the Global Fund for AIDS, Tuberculosis and Malaria and The Carter Center. Previous work demonstrated that mass net distribution in 2010 in the two-state area was associated with disappearance of *W. bancrofti* infections in mosquitoes.^11^ The most recent (2015) national Malaria Indicator Survey (MIS) found that approximately 77% of households in the two states reported having at least one ITN or LLIN.^17^ High net coverage provides mosquitocidal and barrier protection to prevent LF transmission recrudescence or reintroduction after the halt of MDA.

These results also suggest that current stop-MDA guidelines appear sufficient to achieve transmission interruption breakpoints in settings such as Plateau and Nasarawa, that is, areas where *Anopheles* is the main vector, high-coverage MDA is achieved, and bed nets are widespread. Stop-MDA thresholds should ensure a high probability of achieving transmission breakpoints, whereas at the same time avoiding unnecessary and costly rounds of MDA in a parasite-free population. The 2011 TAS guidelines acknowledge that “the precise threshold below which infection cannot be sustained has not been defined except in specific settings” meaning that current thresholds represent tentative indicators.^3^ In Plateau and Nasarawa, we observed a steady decline in antigen prevalence among children aged 6–7 years from stop-MDA surveys (1.0% in the 10 LGAs that qualified to stop in 2007–2008 and 0.4% among children in the 21 LGAs that qualified to stop in 2012) to 0.0% across all 30 LGAs in TAS-2 (2014–2015), and to zero CFA-positive children at TAS-3 (2016–2017). Although comparison of prevalence estimates between TASs should be carried out with caution because of the random selection of survey clusters in each TAS, these results indicate a rapid decline in the LF incidence following the halt of MDA. However, other studies indicate that stop-MDA thresholds may not lead to interruption of transmission in all settings. Studies from American Samoa,^18,19^ Sri Lanka,^20,21^ Zanzibar,^22^ and unpublished reports from Haiti (J. F. Lemoine, personal communication) reveal sustained or even increased *W. bancrofti* transmission after successfully passing one or more TASs. A common feature of these settings is transmission by non-anopheline vectors: *Culex quinquefasciatus* in Sri Lanka, Zanzibar, and Haiti, and the highly efficient *Aedes polynesiensis* in American Samoa. Previous models have predicted unique transmission breakpoints for LF vectors, with median mf breakpoints calculated at 0.2% for culicines and 0.8% for anophelines.^23^ Current TAS cutoffs are calibrated to < 2% antigen prevalence or < 1% mf prevalence for *Culex* and *Anopheles* mosquitoes and < 1% antigen prevalence or 0.5% mf prevalence for *Aedes*. The emerging body of evidence suggests that, although suitable for *Anopheles*-transmission areas, current criteria may need to be revised for *Culex-* and *Aedes*-transmission areas.

Another explanation for sustained or recrudescent transmission in areas passing TASs is incident transmission among adult community members not detected via TASs. This may be due to differences in risk factors, treatment coverage, survey participation, or a combination of factors between adults and children, who are the target population for TASs. Thus, claims of transmission interruption in Plateau and Nasarawa should be treated with caution until confirmed. Recent studies suggest that community-wide testing of children and adults is more sensitive for detecting incident transmission compared with traditional TASs of primary school–aged children.^19,24^ Another confounding factor is that children in many LF-endemic areas are treated with albendazole for the control of soil-transmitted helminths (STHs), oftentimes separately from MDA for LF. In Plateau and Nasarawa, annual community-based albendazole treatments have taken place since 2000. Longitudinal data show that semi-annual albendazole treatment reduced antigen prevalence, mf prevalence, and mf density among microfilaremic individuals.^25^ By receiving one dose of albendazole annually for STH and one dose of albendazole–ivermectin annually for LF for 13 years, reduction of *W. bancrofti* infections may have been accelerated in school-aged populations and not reflective of community-wide transmission rates. Thus, although no antigen-positive children were detected in TAS-3, we cannot exclude the possibility of undetected LF transmission in Plateau and Nasarawa based on TAS results alone. Studies comparing LF antigen prevalence between TAS age-groups and community members aged at least 2 years have been conducted in the area and will be reported separately (Noland et al., in preparation).

Despite passing TAS-3, it is important to continue PTS in Plateau and Nasarawa until all IUs within Nigeria achieve interruption of transmission. In acknowledging TAS-3 survey results, the Nigerian FMOH recommended three activities that we fully endorse: 1) continue PTS especially in hard-to-reach areas and LGAs bordering other LF-endemic states; 2) strengthen integrated vector management through continued promotion of LLINs; 3) support MMDP. Specific methodologies for conducting PTS in EUs that have passed TAS-3 or countries that have achieved validation have not been standardized by the WHO.^14^ Potential options include additional TASs, follow-up of antigen-positive clusters identified in TASs, continued sentinel and spot-check site monitoring, integrating LF antigen or serological testing within large-scale cross-sectional surveys such as MIS surveys, and xenomonitoring, which Togo piloted in operational research studies during post-validation surveillance.^26^ To date, PTS in Plateau and Nasarawa has been limited to operational research studies aforementioned testing both children and adults in selected LGAs. Follow-up foci investigation based on CFA-positive individuals in TAS-2 was not carried out as this was beyond the approved survey protocol (TAS-3 protocols did include options for follow-up investigation, but no CFA-positive samples were identified). Other surveillance activities have not been conducted mainly because of financial limitations, as core funding agencies currently do not provide support for LF programmatic activities beyond TAS-3. This leaves areas such as Plateau and Nasarawa unable to continue PTS and susceptible to undetected recrudescence or importation from neighboring states that continue to experience incident transmission. To support continued promotion of LLINs, the Carter Center has assisted with the distribution of 79,647 LLINs since 2017, specifically targeted to cross-border areas in Plateau and Nasarawa.

Morbidity management and disability prevention is a key pillar of the GPELF and a required element for validation of elimination as a public health problem. Yet, funding for MMDP activities also is scarce. Nonetheless, The Carter Center has long-promoted MMDP activities in Plateau and Nasarawa, beginning with burden estimate surveys conducted during LF mapping in selected LGAs^27^ and through self-report of lymphedema or hydrocele during household visits by community drug distributors, a pilot mass surgery campaign for hydrocele patients,^28^ and Hope Clubs that provide lymphedema management and social support. Recent support from the Izumi Foundation will enable the expansion of health facilities to provide the basic care package throughout Plateau and Nasarawa.

These surveys highlight the complexities of conducting LF PTS in onchocerciasis co-endemic areas. Of the 30 LGAs in Plateau and Nasarawa, 12 were classified as hyper-/meso-endemic and under ivermectin MDA throughout the course of TASs. The first complication is EU formation, which gave priority to LF epidemiological similarity rather than onchocerciasis co-endemicity status. This resulted in a mixture of hyper-/meso-endemic areas and hypo-/non-endemic areas for most EUs. A second complication is the timing of PTS activities. Ivermectin monotherapy significantly reduces the number of *W. bancrofti* mf in circulation.^29,30^ Therefore, can areas that continue to provide ivermectin truly be considered as in the “PTS” phase? From the onchocerciasis perspective, WHO guidelines indicate that PTS for onchocerciasis cannot begin until LF MDA stops in co-endemic areas.^31^ Although we believe that LF surveillance activities along the PTS timeline are still warranted to detect recrudescence or importation in areas providing ivermectin after qualifying to stop LF MDA, we argue that additional surveys—ideally integrated in nature—need to be conducted after the halt of both albendazole and ivermectin in formerly co-endemic areas to substantiate claims of LF transmission interruption in the absence of interventions.

We identify the following limitations in these surveys. A total of eight schools in TAS-2 and 13 schools in TAS-3 were not visited because of insecurity at the time of sampling. Areas of central Nigeria periodically experience clashes between pastoralists and settled populations over land use and other issues. Such areas are of particular concern to LF elimination for several reasons: 1) absence of up-to-date survey data, 2) they were potentially inaccessible during MDA and thus may harbor pockets of active transmission, 3) movement of pastoralist in and out of the IU poses a significant risk for parasite introduction. Therefore, PTS should prioritize geographic areas inaccessible during TASs as well as outreach to mobile populations who move through the region. A second limitation is the aggregation of multiple IUs into EUs. Ideally, each of the 30 LGAs in the study area, each as an IU, would function as an individual EU to provide LGA-specific results. However, this was financially and programmatically untenable. The present surveys followed the EU configurations from TAS-1^10^ to enable EU consistency between surveys. Transmission assessment surveys-1 EU groupings were made based on epidemiological similarity and TAS population limits, which comply with WHO recommendations for EU formation.^3^ However, grouping multiple IUs together may have further exacerbated the predicted limited ability of TAS to detect microfoci of transmission.^32^ A third limitation is that procedures for selecting children at each school during 2014–2016 surveys differed slightly from recommended TAS procedures. Whereas the TAS procedures generate a fixed sampling fraction across all schools in an EU to yield equal probabilities of selection for each child, our protocol selected a similar number of children at each school to minimize confusion for survey teams. Although the equal probability of selection assumption for TAS was not met, the pass/fail outcome for any EU was not affected. In addition, our procedures targeted approximately 50% more clusters (45 schools versus 30 schools) than would be recommended by *Survey Sample Builder*,^33^ meaning that our results likely are more representative compared with a conventional TAS and, therefore, more conservative in its determinations. Our 2017 surveys used a fixed sampling fraction as recommended by TAS guidelines but still sampled a larger number of clusters.

In conclusion, these results indicate that LF transmission remains below sustainable transmission levels in Plateau and Nasarawa, Nigeria, and suggest that elimination of transmission has been achieved—though further studies are required to substantiate this claim.
